# Relationship Between Anabolic–Androgenic Steroid Use, Aggression, and Narcissism in Male Bodybuilders

**DOI:** 10.3390/medicina61020241

**Published:** 2025-01-30

**Authors:** Eren Ceto, Pembe Hare Yigitoglu, Hasan Ulas Yavuz

**Affiliations:** 1 Faculty of Sport Sciences, Near East University, Nicosia 99250, Cyprus; eren.ceto@neu.edu.tr; 2 Department of Physical Medicine and Rehabilitation, Nicosia State Hospital, Nicosia 99250, Cyprus; pembe.hare@hotmail.com

**Keywords:** anabolic steroids, aggression, narcissism, anger

## Abstract

*Background and Objectives:* The use of anabolic–androgenic steroids (AASs) by competitive and recreational athletes has been studied and well documented. There are numerous studies showing its effects on personality traits and risky behaviors like aggression. The relationship between AAS use, aggression, and narcissism is complex and intricate. We examined this relationship in male bodybuilders who use AASs. *Materials and Methods:* A total of 319 healthy subjects aged 18–44 years (33.4 ± 9.4) who have been regularly training at bodybuilding for at least 3 years participated voluntarily in the study and completed a demographic data inventory, the Five-Factor Narcissism Inventory Short Form (FFNI-SF), and the Buss–Perry Aggression Scale anonymously. Demographic data were given as percentages, comparisons of aggression and narcissism scores according to AAS use were performed by using an independent sample *t* test, and effects of narcissism and aggression levels on AAS use was assessed by using logistic regression analysis. All analyses were performed by using SPSS Statistics 22.0. *Results:* Results showed that AAS users had significantly higher scores on the overall FFNI-SF Scale (*p* < 0.001) and all sub-dimensions of narcissism (*p* < 0.001) and on the overall Buss–Perry Aggression Scale (*p* < 0.001) and all sub-dimensions of aggression (*p* < 0.001). It was also shown that there were significant and positive correlations between the FFNI-SF overall score (*p* < 0.001) and both the vulnerable narcissism and grandiose narcissism sub-dimensions (*p* < 0.001) and the scores of the Buss–Perry Aggression Scale (*p* < 0.001), physical aggression (*p* < 0.001), anger (*p* < 0.001), hostility (*p* < 0.001), and verbal aggression (*p* < 0.001) sub-dimensions. *Conclusions:* These results show a strong relation between AAS use, narcissism, and aggression in bodybuilders. However, it is not clear whether AAS use leads to aggression and narcissism or whether narcissistic and/or aggressive people tend to use AASs. Furthermore, including a lot of potential third variables shows that it does not have to be either one or the other way around. There is a need to conduct future studies to determine this causality.

## 1. Introduction

The use of anabolic–androgenic steroids (AASs), which are various derivatives of testosterone, the male sex hormone, for enhancing appearance and bodybuilding has become increasingly common. For many years, elite athletes from different sports have used AASs to improve athletic performance. Today, the majority of AAS users are not competitive athletes, but individuals who want to look more muscular and fitter [1].

It has been reported that more than half a million high school students in the United States of America use AASs outside of medical indications [2]. It has also been reported that AAS use is positively associated with the use of alcohol, drugs, and legal performance-enhancing substances, but the association with tobacco and cannabis use is not as clear [3]. AAS use is higher in athletic students compared with non-athletes. Among students, those who are interested in American football, wrestling, weightlifting, and especially bodybuilding, are more likely to use AASs than other students [4]. AASs are often used in ’cycles’, which refers to the use of multiple doses of AASs over a specific period of time, stopping use for a specific period of time, and then starting again. The duration of the cycles shows diversity according to the AAS that is used, personal preferences, objectives to be achieved at the end of the cycle, and previous experiences of the person [5]. Each cycle might have a different duration even if it is performed by the same person.

The increasing popularity of AAS usage creates a serious concern among physicians, who are aware of the potential side effects of AASs [1,2]. AASs have anabolic (muscle building) and androgenic (masculinizing) properties. The AAS groups are modified to enhance anabolic rather than androgenic properties. Anabolic effects such as protein synthesis, muscle growth, and erythropoiesis are preferred by athletes and have positive effects on muscle growth, lean muscle mass, and endurance [6].

Despite its positive effects on athletic performance and aesthetics [6,7], negative emotions such as lack of impulse control, aggression, hostility, and violent behavior have been reported [8,9,10] with AAS use. In a recent meta-analysis, 12 randomized controlled trials were evaluated, and it was reported that AAS use increased self-reported aggression [11]. However, this meta-analysis focused on nonphysical aggression like increased hostility and verbal aggression. It is imperative to acknowledge that experiencing aggression does not necessarily lead to violence and that aggression and violence are not synonymous.

There is mounting evidence indicating a correlation between the use of anabolic–androgenic steroids (AASs) and various forms of violence, including physical altercations, violent crimes, and intimate partner violence [12]. Another recent meta-analysis revealed a modest yet statistically significant correlation between AAS use and interpersonal violence. But still, the direction of this relationship remains uncertain, as the majority of studies have not elucidated whether AAS use preceded or followed the occurrence of violent behavior [13].

It is important to understand the relationship between AASs and violence because violence linked to substance abuse has a significant impact not only on the person but also on society. At the individual level, aggressive behaviors have been demonstrated to exert a detrimental influence on mental well-being, often giving rise to conditions such as depression and anxiety. At the community level, these behaviors can engender social discord and incite social conflicts as well as physical violence. The erosion of trust and mutual respect among members that characterizes aggressive behaviors has been shown to contribute to the escalation of social conflicts and the emergence of polarization, which can ultimately result in physical violence [14].

However, due to inconsistencies in the findings across studies, it remains unclear whether unobserved patterns (i.e., those not directly measured or detected) of aggression and psychological distress exist among AAS users [15]. A preliminary study suggested that AAS users exhibit higher levels of pathological narcissism and significantly lower levels of empathy. However, the directionality of this relationship is still uncertain—whether AAS use leads to narcissism or whether narcissism predisposes individuals to AAS use requires further investigation [16]. Recent meta-analyses have confirmed a significant relationship between narcissism and aggression [17,18], but these studies also highlight that provocation plays a key role in this link. Narcissistic individuals tend to become more aggressive when provoked, and narcissism itself is a significant risk factor for aggression and violence [18]. The narcissistic anger theory posits that narcissistic individuals experience more intense emotional reactions to interpersonal distress, marked by emotional volatility and heightened emotional intensity [19]. These individuals are particularly prone to negative emotions such as shame, anger, and anxiety in the face of threat, which may lead to aggressive behaviors [20].

The Buss–Perry Aggression Scale and Five-Factor Narcissism Inventory were selected to measure aggression and narcissism. The Buss–Perry Aggression Scale provides a multidimensional measure of aggression and allows the effects of steroid use on different types of aggression to be examined. Similarly, the Five-Factor Narcissism Inventory provides a multidimensional measure of narcissism and allows a more detailed assessment of narcissistic tendencies in steroid users.

As can be understood in light of all this information, there is a complex and intricate relationship between AAS use, aggression, and narcissism. While previous studies such as the meta-analysis by Chegeni et al. [11] have systematically examined the relationship between AAS use and aggression, the role of personality traits like narcissism in this dynamic has not been adequately addressed. Our study uniquely explores the interplay among anabolic steroid use, narcissism, and aggression in male bodybuilders, offering novel insights into the psychological and behavioral patterns associated with steroid use.

## 2. Materials and Methods

### 2.1. Participants

A total of 319 healthy subjects aged 18–44 years (33.4 ± 9.4) who have been regularly training at bodybuilding for at least 3 years participated voluntarily in the study.

Individuals under 18 years of age or over 45 years of age; individuals who have been training for fewer than 3 years, and individuals with a diagnosis of psychiatric disorder were excluded from the study.

Individuals who have been training for 3 years or more but not training regularly were excluded from the study since including such individuals could lead to misinterpretation of results by blending outcomes of regular and irregular training practices of bodybuilding.

### 2.2. Study Design

This study was performed as a cross-sectional survey study in 10 different fitness centers in Nicosia, Cyprus in 2021–2022. A total of 21 certified gyms serving in the Nicosia were identified, and the 10 gyms with the highest number of registered members from different districts were identified and included in the study. Gyms were visited on different days between 17:00 and 19:00. Information about the study was given, and consent forms were signed by those who agreed to participate. Afterwards, the participants were asked to fill out the questionnaires, and the completed forms were collected. The names of the participants were kept anonymous throughout the study, and the data collected were not revealed to anyone outside of the study.

### 2.3. Measures

Participants were assessed by a sociodemographic data form designed by the researcher that included questions about demographic properties; training information, including number of cycles per year and years of training; and AAS usage. As demographic characteristics, the participants’ self-reported education level (high school or below, university, postgraduate), marital status (married, single, widowed, or divorced) and income (good, medium, poor) were examined.

Then, they were assessed by the short form of the Five-Factor Narcissism Inventory the and Buss–Perry Aggression Questionnaire.

The Five-Factor Narcissism Inventory (FFNI) was developed by Glover et al. [21] to assess both vulnerable and grandiose variants of narcissism. FFNI is a 148-item self-report inventory of 15 traits designed to assess the basic elements of narcissism from the perspective of a 5-factor model. The short form of the FFNI (FFNI-SF), which has a 60-item self-report inventory, was developed by Sherman et al. [22], and the Turkish version of the FFNI-SF was developed by Eksi [23]. It consists of 60 items loaded on 15 factors: acclaim seeking, arrogance, authoritativeness, distrust, entitlement, exhibitionism, exploitativeness, grandiose fantasies, indifference, lack of empathy, manipulativeness, need for admiration, reactive anger, shame, and thrill-seeking. The scale uses a 5-point Likert-type scale ranging from 1 (strongly disagree) to 5 (strongly agree). Vulnerable narcissism is the sum of need for admiration, reactive anger, and shame while grandiose narcissism is the sum of the remaining scales [22].

The Buss–Perry Aggression Questionnaire (BAQ) was developed in 1992 to assess levels of aggression. It consists of 29 items and 4 subscales: physical aggression (items 2, 5, 8, 11, 13, 16, 22, 25, 29), verbal aggression (items 4, 6, 14, 21, 27), anger (items 1, 9, 12, 18, 19, 23, 28), and hostility (items 3, 7, 10, 15, 17, 20, 24, 26) [24]. Each item is rated on a Likert scale ranging from 1 (strongly disagree) to 5 (strongly agree). The Turkish version of the Buss–Perry Aggression Questionnaire (BAQ) was developed by Madran [25].

### 2.4. Statistical Analysis

The Kolmogorov–Smirnov test was applied to assess the normality of the data, and the data followed a normal distribution. Demographic data were given as percentages, comparisons of aggression and narcissism scores according to AAS use were performed by using independent sample *t* test, effects of narcissism and aggression levels on AAS use were assessed by using logistic regression analysis. Correlations between athletes’ Five-Factor Narcissism Inventory Short Form and Buss–Perry Aggression Scale scores were assessed using correlation analysis. Since the data set was normally distributed, the Pearson product–moment correlation coefficient was used for correlations. All analyses were performed by using SPSS Statistics 22.0. Statistical significance was established at *p* < 0.05. Bonferroni correction was applied for multiple comparisons, and the significance level was set as accordingly.

### 2.5. Ethics

After the participants were verbally informed about the details of the study, a consent form was distributed to and signed by the participants. The study was conducted in accordance with the principles of the Helsinki Declaration. The study protocol was approved by the Near East University Ethics Committee (YDU/2021/88-1278).

## 3. Results

The results of this study provide insight into the relationship between AAS use, aggression, and narcissism, highlighting the patterns and associations observed among the participants. Seventy-eight participants reported having used AASs at some point, while seventy-six indicated that they were currently using AASs (either during or between cycles). Sociodemographic data on AAS use were presented for all participants, while psychological test data were analyzed specifically for those who reported being current users in order to capture the immediate effects of AAS usage.

As shown in Table 1, the results of the chi-square test indicated that there was no significant difference in AAS usage with respect to the participants’ education level and accommodation. However significant differences were observed in marital status and income levels between AAS users and non-users.

Comparison of narcissism and aggression scores among athletes based on AAS usage are presented in Table 2. Significant differences were observed across all dimensions, with AAS users scoring higher on both narcissism (vulnerable and grandiose subtypes) and aggression (physical aggression, anger, hostility, and verbal aggression).

The scores of the athletes who used AASs on the overall FFNI-SF scale and its sub-dimensions, vulnerable narcissism and grandiose narcissism, were found to be statistically significantly higher than the scores of the athletes who did not use AASs.

The physical aggression, anger, hostility, and verbal aggression scores and the Buss–Perry Aggression Scale general scores of athletes who used AASs were found to be statistically significantly higher than the scores of athletes who did not use AASs.

Comparison of narcissism and aggression scores based on duration of AAS use among athletes are shown in Table 3. The findings show significant differences in anger, hostility, and overall aggression scores, with longer durations of AAS use generally associated with higher scores. No significant variations were observed in narcissism scores across groups.

Buss–Perry Aggression Scale general scores and anger scores of athletes who used AASs for 1–12 months were significantly lower than the scores of athletes who used AASs for 13–24 months and for 25 months or more.

The hostility scores of the participants who used AASs for 13–24 months were significantly higher than the scores of the athletes who used AASs for 1–12 months and those who used AASs for 25 months or more.

Comparison of psychological and aggression scores across different frequencies of AAS use in athletes are presented in Table 4. Significant differences were identified in several subscales, including grandiose narcissism, physical aggression, anger, and hostility. 

The overall FFNI-SF scale and the grandiose narcissism sub-dimension scores of the athletes who used AASs for 1 cycle per year were statistically significantly lower than those for the ones who used AASs for 2 cycles per year and 3 cycles per year.

A total of 24 participants (31.6%) stated that they had been doing 1 cycle per year, 37 participants (48.7%) for 2 cycles per year, and 15 participants (19.7%) 3 cycles per year.

The physical aggression and Buss–Perry Aggression Scale overall scores of the athletes who used 1 cycle of AASs per year were significantly lower than those of the athletes who used 2 cycles of AASs per year and 3 cycles of AASs per year, while the anger scores of the athletes who used 2 cycles of AASs per year were significantly higher than those of the athletes who used 1 cycle of AASs per year. The hostility scores of the athletes who used 3 cycles of AASs per year were significantly higher than the hostility scores of the athletes who used 1 or 2 cycles of AASs per year.

Table 5 presents the results of a binary logistic regression analysis, evaluating the impact of scores from the FFNI-Short Form and Buss–Perry Aggression Scale on the likelihood of AAS use.

It was determined that the FFNI-SF scores had a statistically significant effect on the odds of using AASs (*p* < 0.05). Accordingly, if the athletes’ scores on the FFNI-SF scale increase by 1 point, the odds of using AASs increase by 2%.

It was found that Buss–Perry Aggression Scale scores had a statistically significant effect on the risk of using AASs (*p* < 0.05). An increase of 1 point in the scores of the athletes in the Buss–Perry Aggression Scale increases the risk of using AASs by 7%.

Table 6 shows that there were statistically significant and positive correlations between the scores of the participants from the FFNI-SF overall score, and the vulnerable narcissism and grandiose narcissism sub-dimensions, and the scores from the Buss–Perry Aggression Scale and the physical aggression, anger, hostility, and verbal aggression sub-dimensions.

## 4. Discussion

The relationships between anabolic–androgenic steroid usage, narcissism, and aggression levels of 319 men training in bodybuilding was examined in the present study. The results showed that AAS users had significantly higher scores on the overall FFNI-SF scale (*p* < 0.001) and all sub-dimensions of narcissism (vulnerable narcissism and grandiose narcissism) (*p* < 0.001) and the overall Buss–Perry Aggression Scale (*p* < 0.001) and all sub-dimensions of aggression (physical aggression, anger, hostility, and verbal aggression) (*p* < 0.001) compared with non-users.

The higher aggression score among AAS users compared with non-users is consistent with previous studies [26,27,28,29,30]. Melloni and Ricci hypothesized that testosterone may increase aggression by reducing activity in the orbitofrontal cortex [31]. In the literature, it is well established that anabolic–androgenic steroids (AASs) can easily cross the blood–brain barrier and interact with androgen receptors in the central nervous system [32]. Since the orbitofrontal cortex is responsible for regulating emotional responses and executive control [33,34,35], dysfunctions in this area have been linked to aggression and violent behavior [36]. In line with these findings, Hauger et al. [9] reported thinning of the orbitofrontal cortex and impaired behavioral regulation in AAS users compared with non-users [37].

The relationship between AASs and aggressive behavior is more evident in animal studies, where it was observed that AAS administration resulted in increased aggression across all studies [38,39,40]. However, the relationship is more multifaceted in humans, and it can be influenced not only by individual factors but also by environmental factors [26]. Geniole et al. suggested that the role of AASs in aggression might be affected by personality traits such as increased dominance and decreased self-control [41]. In a recent study by Hauger et al. (2021) [26], the association between AAS use, aggression, and violence was studied. The researchers examined the relationship between the use of anabolic–androgenic steroids (AASs) and three key factors: dependence, aggression, and violence. To assess the levels of aggression, they employed the Buss–Perry Aggression Questionnaire. The study employed a three-group design comprising a non-user control group, a group of dependent users, and a group of non-dependent users. The findings indicated that dependent users exhibited significantly elevated levels of aggression relative to non-users and non-dependent users. The researchers highlighted the potential role of antisocial personality traits as key mediators of aggressive behavior.

There was no difference in narcissism and its sub-dimension scores between the groups with respect to the duration of the AAS use (*p* > 0.5). Nevertheless, it was noted that there were discrepancies between the groups with regard to the overall aggression scores (*p* = 0.01) as well as the anger (*p* = 0.001) and hostility (*p* = 0.000) sub-dimensions. This may be an understandable distinction, given that narcissism is a personality trait, whereas aggression is more closely related to behavior. It is noteworthy that scores for anger, hostility, and overall aggression demonstrated an increase over a duration of 13–24 months in comparison with a duration of 1–12 months. However, there was a subsequent tendency for a decrease in scores for these variables over a duration of >24 months. It can be speculated that the initial response to AASs may result in the development of heightened aggression, which may subsequently lead to the formation of tolerance in response to chronic AAS exposure. However, this approach is not aligned with the findings of a previous study [26], which indicated that a higher lifetime exposure to AASs may be associated with higher levels of aggression. In the present study, the sample exclusively comprised bodybuilders, whereas the aforementioned study encompassed individuals from a range of sports disciplines (bodybuilding, weightlifting, combat sports, endurance sports, and ball sports) who engaged in weight training. Additionally, the participants in that study were categorized into AAS-dependent and non-dependent groups, with findings indicating higher levels of aggression among those who were AAS-dependent. However, the present study did not consider dependency on AAS as a criterion. Additionally, other confounding variables, such as the frequency and duration of AAS use or the participants’ baseline aggression levels, could influence the outcomes. Further research, including a more detailed exploration of these factors, would be valuable in resolving such inconsistencies.

The current study revealed that the frequency of AAS use (1, 2, or 3 cycles per year) was associated with higher scores on measures of grandiose narcissism (*p* = 0.021) and overall narcissism (*p* = 0.014), as well as on measures of physical aggression (*p* = 0.041), anger (*p* = 0.001), hostility (*p* = 0.000) and overall aggression (*p* = 0.002), with increasing frequency. As the number of cycles per year increases, there is a tendency for an increase in all scores. The AAS cycles might show variation according to the composition and content of the AASs, the duration of the cycles, and the doses. Since these variables were not determined in the present study, it is possible only to speculate that continuous exposure to AASs might be the reason for this tendency.

The present study demonstrated that the FFNI-SF and Buss–Perry Aggression Scale had a notable impact on the likelihood of using AASs. Consequently, an increase of 1 point on the FFNI-SF Scale is associated with a 2% increase in the likelihood of using AASs (*p* = 0.034), while a 1-point increase on the Buss–Perry Aggression Scale is linked to a 7% increase in the probability of using AASs (*p* = 0.000) (R^2^ = 0.440, accuracy 85.6%, 95% confidence interval). The R^2^ value indicates that 44% of the variance in AAS use can be explained by aggression and narcissism levels. The higher aggression scores, particularly in the physical aggression and anger subscales, among AAS users suggest that steroid use may amplify tendencies toward confrontational and impulsive behaviors. Similarly, elevated narcissism scores in both grandiose and vulnerable dimensions highlight the potential role of personality traits in driving the motivation for AAS use. These findings suggest that individuals who score higher on aggression and narcissism scales may be at greater risk of AAS use, which has significant implications for identifying and supporting at-risk populations. For example, psychological screening tools incorporating these traits could aid in early intervention programs targeting athletes or bodybuilders. However, it is important to note that while the relationship between these variables is robust, causality cannot be inferred from these findings. The unexplained variance indicates that other contributing factors, such as social or environmental influences, should be explored in future studies to develop a more comprehensive understanding of AAS use. Previous studies have indicated that individuals who use AASs exhibit a higher prevalence of severe narcissistic personality traits when compared with those who do not use AASs [16,42]. It was proposed that preexisting personality disorders, such as narcissism, among athletes may contribute to an increased proclivity to the use of AASs [16,43].

Although aggression has been reported as an adverse effect of AASs, supra-physiological doses of AAS administration to healthy male subjects have failed to demonstrate such an association [28,44]. In addition, AAS users have been reported to have higher aggressive tendencies prior to AAS use [26,45]. Sagoe et al. reported that aggressive adolescents had higher intentions to use AASs than non-aggressive adolescents, emphasizing that aggression is a risk for AAS use in adolescents [45].

It might not seem easy to determine whether AAS use leads to aggression and narcissism or whether narcissistic or/and aggressive people tend to use AASs. The nature-versus-nurture debate is a long-standing and contentious topic among neuroscientists. It concerns the extent to which genetic and environmental factors influence human behavioral differences [46]. It appears that neurobiological irregularities are more closely associated with aggressive behavior than an unfavorable upbringing. However, in the majority of instances, the combined influence of these two factors is necessary to elicit such conduct [47]. Similarly, it seems that narcissistic personality disorder has a multifactorial etiology and pathogenesis, with a multitude of mechanisms linked to each area of dysfunction. There is evidence from studies that genetic predisposition may play a role, with findings indicating that there are inherited variations associated with hypersensitivity, strong and aggressive drive, low anxiety or frustration tolerance, and defects in affect regulation [48].

Additionally, the results demonstrated a notable and positive correlation between the overall score on the FFNI-SF and both the vulnerable narcissism and grandiose narcissism sub-dimensions, as well as the scores on the Buss–Perry Aggression Scale, encompassing physical aggression, anger, hostility, and verbal aggression.

A recent meta-analysis that assessed 437 studies comprising a total of 123,043 participants reported that narcissism showed significant correlations with both aggression and violence [18]. It was also shown that narcissism, whether pathological or normal, is still related to aggression, with all three sub-dimensions being related to all forms of aggression. Our findings are consistent with these results. They have also emphasized that narcissistic individuals are prone to aggression under provocation, suggesting that narcissism is an important risk factor for aggression and violence [18].

Since AASs represent a wide variety of chemicals with different anabolic and androgenic effects, generalizing about a group of chemicals as if they all have the same or very similar effects may mislead the results and interpretation of the results. Evaluation of individual AASs in future longitudinal studies may clarify this relationship.

## 5. Limitations

The present study does present with some limitations. As the study was cross-sectional in nature, the determination of causality is not appropriate. Longitudinal studies will be needed to elucidate the causality.

Although all questionnaires were completely anonymous, as AAS use is a sensitive issue for many athletes, the social desirability bias risk still existed.

The timing of data collection could introduce bias, as it excluded some demographics that go to the gym at different times.

It was questioned whether the participants use AASs or not, for how long they use them, and how many cycles they have in a year. But the content, dosage, and duration of each cycle, which might affect the results and interpretation of the study, were not assessed in the study.

Only male bodybuilders participated in the study, which makes it difficult to apply these findings to female AAS users or AAS users in any other sports.

Participants who have been regularly training for at least three years were included in the study, and this was determined according to the self-reports of the participants. Since all participants were volunteers and there were no specific criteria to verify their training characteristics, this ease of participation may cause a relatively large margin of error.

Another limitation of this study is the exclusion of irregular trainees, who represent a significant subgroup of the bodybuilding community. This choice was made to ensure sample homogeneity, but it may limit the generalizability of our findings to the broader population of bodybuilders.

Finally, there may be various contextual factors that were not assessed in this study but that may influence the relationship between AAS use, aggression, and narcissism.

## 6. Conclusions

In conclusion, the results show a strong correlation between AAS use, narcissism, and aggression in male bodybuilders. However, it is not clear whether AAS use leads to aggression and narcissism or whether narcissistic and/or aggressive people tend to use AASs. Furthermore, since this association is complicated, including a lot of potential confounding variables, it does not have to be necessarily either one or the other way. Future studies will be needed to assess this causality.

## Figures and Tables

**Table 1:** Sociodemographic characteristics and training profiles of athletes based on AAS usage. This table summarizes the sociodemographic characteristics and training profiles of athletes categorized by their AAS usage. Key findings indicate significant differences in marital status and income levels between AAS users and non-users. The chi-square test, adjusted with Bonferroni correction, was used for statistical analysis, setting a significance level of 0.0167. Limitations include potential reporting bias and the inability to establish causation due to the cross-sectional design.

		AAS USAGE				*p*
		Users		Non-Users		
		*n* (78)	% (24.5)	*n* (241)	% (75.5)	
Age (years)		29.7 ± 6.7		34.6 ± 9.9		0.058
Duration of training (years)		8.1 ± 5.1		8.1 ± 6.6		0.114
Education	High school or below	24	30.8%	49	20.3%	0.061
	University	39	50.0%	118	49.0%	
	Postgraduate	15	19.2%	74	30.7%	
Marital status	Married	37	47.4%	133	55.2%	0.021
	Single	40	51.3%	91	37.8%	
	Divorced/widowed	1	1.3%	17	7.1%	
Income	Low	49	62.8%	104	43.2%	0.032
	Average	29	37.2%	120	49.8%	
	High	0	0.0%	17	7.1%	
Accommodation	Alone	62	79.5%	195	80.9%	0.133
	With family (spouse/partner, children)	14	17.9%	46	19.1%	
	With parents	2	2.6%	0	0.0%	

Chi-square test with Bonferroni correction was applied for multiple comparisons, and the significance level was set as 0.0167 instead of 0.05.

**Table 2:** Comparison of narcissism and aggression scores among athletes based on AAS usage. This table presents the comparison of narcissism and aggression scores between athletes who use AASs and those who do not. Significant differences were observed across all dimensions, with AAS users scoring higher on both narcissism (vulnerable and grandiose subtypes) and aggression (physical aggression, anger, hostility, and verbal aggression). These findings highlight the psychological profile differences associated with AAS usage. Independent sample *t*-tests were conducted, and statistical significance was set at *p* < 0.05.

	AAS Usage	*n*	$\bar{x}$	s	*t*	*p*
Vulnerable Narcissism	Yes	76	38.71	6.20	6.282	0.000 *
	No	243	33.06	7.03		
Grandiose Narcissism	Yes	76	154.36	21.97	6.396	0.000 *
	No	243	135.79	22.13		
Narcissism Scale	Yes	76	193.07	24.96	7.237	0.000 *
	No	243	168.84	25.62		
Physical Aggression	Yes	76	31.92	6.43	12.420	0.000 *
	No	243	20.84	6.90		
Anger	Yes	76	24.54	5.04	9.132	0.000 *
	No	243	17.88	5.70		
Hostility	Yes	76	27.38	5.43	9.515	0.000 *
	No	243	20.66	5.36		
Verbal Aggression	Yes	76	15.91	3.65	3,318	0.001 *
	No	243	14.48	3.16		
Buss–Perry Aggression Scale	Yes	76	99.75	17.77	10.812	0.000 *
	No	243	73.86	18.36		

* *p* < 0.05, independent sample *t* test.

**Table 3:** Comparison of narcissism and aggression scores based on duration of AAS use among athletes. This table presents a comparison of the narcissism and aggression scores among athletes categorized by the duration of their AAS use. A total of 27 participants (35.5%) reported AAS use for 1-12 months, 26 (34.2%) for 13–24 months, and 23 (30.3%) for >24 months. The findings show significant differences in anger, hostility, and overall aggression scores, with longer durations of AAS use generally associated with higher scores. No significant variations were observed in narcissism scores across groups. ANOVA with Bonferroni correction was applied for multiple comparisons, setting the significance level at 0.0167. The cross-sectional design and small sample size in the subgroups may limit the generalizability of these findings.

	Duration	*n*	$\bar{x}$	s	*p*	Difference
Vulnerable Narcissism	1–12 months	27	36.00	8.44	0.054	
	13–24 months	26	40.19	3.32		
	>24 months	23	40.22	4.52		
Grandiose Narcissism	1–12 months	27	154.19	21.59	0.972	
	13–24 months	26	155.15	17.42		
	>24 months	23	153.65	27.37		
Narcissism Scale	1–12 months	27	190.19	26.14	0.735	
	13–24 months	26	195.35	19.67		
	>24 months	23	193.87	29.33		
Physical Aggression	1–12 months	27	30.22	6.94	0.142	
	13–24 months	26	32.35	5.53		
	>24 months	23	33.43	6.55		
Anger	1–12 months	27	21.96	5.03	0.001 *	1–2
	13–24 months	26	26.46	4.16		1–3
	>24 months	23	25.39	4.87		
Hostility	1–12 months	27	25.00	4.86	0.000 *	1–2
	13–24 months	26	30.65	4.68		2–3
	>24 months	23	26.48	5.20		
Verbal Aggression	1–12 months	27	16.26	3.89	0.162	
	13–24 months	26	15.12	3.23		
	>24 months	23	16.39	3.81		
Buss–Perry Aggression Scale	1–12 months	27	93.44	18.51	0.010 *	1–2
	13–24 months	26	104.58	14.51		1–3
	>24 months	23	101.70	18.76		

ANOVA with Bonferroni correction was applied for multiple comparisons, and the significance level was set at 0.0167 instead of 0.05. * *p* < 0.0167

**Table 4:** Comparison of psychological and aggression scores across different frequencies of AAS use in athletes. This table compares athletes’ scores on the FFNI-Short Form and Buss–Perry Aggression Scale across three frequency groups of AAS use (1, 2, or 3 cycles per year). Significant differences were identified in several subscales, including grandiose narcissism, physical aggression, anger, and hostility (Bonferroni-corrected ANOVA, *p* < 0.0167). These findings suggest that increased frequency of AAS use is associated with elevated aggression and narcissistic traits. However, the results should be interpreted with caution due to the reliance on self-reported data and the relatively small sample size in higher-frequency groups.

	Frequency	*n*	$\bar{x}$	s	*p*	Difference
Vulnerable Narcissism	1 cycle/year	24	36.38	8.68	0.221	
	2 cycle/year	37	39.78	4.57		
	3 cycle/year	15	39.80	3.90		
Grandiose Narcissism	1 cycle/year	24	144.71	23.04	0.021 *	1–2
	2 cycle/year	37	158.03	18.66		1–3
	3 cycle/year	15	160.73	24.10		
Narcissism Scale	1 cycle/year	24	181.08	26.97	0.014 *	1–2
	2 cycle/year	37	197.81	21.80		1–3
	3 cycle/year	15	200.53	23.56		
Physical Aggression	1 cycle/year	24	29.33	7.07	0.041 *	1–2
	2 cycle/year	37	32.57	6.40		1–3
	3 cycle/year	15	34.47	3.80		
Anger	1 cycle/year	24	21.83	5.14	0.001 *	1–2
	2 cycle/year	37	26.16	4.82		
	3 cycle/year	15	24.87	3.66		
Hostility	1 cycle/year	24	25.13	4.52	0.000 *	1–3
	2 cycle/year	37	25.33	4.12		2–3
	3 cycle/year	15	29.68	5.58		
Verbal Aggression	1 cycle/year	24	15.33	4.30	0.260	
	2 cycle/year	37	15.73	3.48		
	3 cycle/year	15	17.27	2.69		
Buss–Perry Aggression Scale	1 cycle/year	24	91.63	19.41	0.002 *	1–2
	2 cycle/year	37	101.93	17.38		1–3
	3 cycle/year	15	104.14	11.25		

ANOVA with Bonferroni correction was applied for multiple comparisons, and the significance level was set at 0.0167 instead of 0.05. * *p* < 0.0167

**Table 5:** The effect of athletes’ FFNI-Short Form and Buss–Perry Aggression Scale scores on their use of AASs. This table presents the results of a binary logistic regression analysis, evaluating the impact of scores from the FFNI-Short Form and Buss–Perry Aggression Scale on the likelihood of AAS use. Higher scores on the Buss–Perry Aggression Scale (β = 0.07, *p* < 0.001) and the FFNI-Short Form (β = 0.02, *p* = 0.034) were associated with increased probability of AAS use. Nagelkerke R^2^ indicates that the model explains 44.0% of the variance, with an accuracy of 85.6%. The findings underscore significant psychological predictors of AAS use, though potential limitations include reliance on self-reported scores and unmeasured confounding factors.

	β	S.E.	Wald	*p*	%95 C.I.	
					Low	High
Five-Factor Narcissism Inventory Short Form	0.02	0.01	4.49	0.034 *	1.00	1.03
Buss–Perry Aggression	0.07	0.01	42.78	0.000 *	1.05	1.09
Constant	−6.24	1.56	15.99	0.000 *		

* *p* < 0.05; Nagelkerke R^2^ = 0.440; accuracy 85.6%; C.I. 95%, confidence interval binary logistic regression.

**Table 6:** Correlations between athletes’ FFNI-Short Form and Buss–Perry Aggression Scale scores. This table illustrates the correlations between dimensions of narcissism (vulnerable and grandiose) and aggression components (physical aggression, anger, hostility, and verbal aggression). Significant positive correlations were observed between grandiose narcissism and physical aggression as well as between vulnerable narcissism and anger. However, the table does not account for potential moderating variables, which may influence these associations.

	Vulnerable Narcissism		Grandiose Narcissism	FFNI-SF Score	Physical Aggression	Anger	Hostility	Verbal Aggression	Buss–Perry Aggression Scale
Vulnerable Narcissism	r	1	0.446	0.645	0.501	0.566	0.609	0.401	0.597
	*p*	-	0.000 *	0.000 *	0.000 *	0.000 *	0.000 *	0.000 *	0.000 *
Grandiose Narcissism	r		1	0.972	0.510	0.414	0.457	0.431	0.517
	*p*			0.000 *	0.000 *	0.000 *	0.000 *	0.000 *	0.000 *
FFNI-SF Score	r			1	0.568	0.503	0.551	0.474	0.600
	*p*				0.000 *	0.000 *	0.000 *	0.000 *	0.000 *
Physical Aggression	r				1	0.815	0.724	0.594	0.926
	*p*					0.000 *	0.000 *	0.000 *	0.000 *
Anger	r					1	0.795	0.620	0.933
	*p*						0.000 *	0.000 *	0.000 *
Hostility	r						1	0.555	0.887
	*p*							0.000 *	0.000 *
Verbal Aggression	r							1	0.727
	*p*								0.000 *
Buss–Perry Aggression Scale	r								1
	*p*								-

* *p* < 0.05. Pearson product–moment correlation coefficient.

## Data Availability

The authors confirm that the data supporting the findings of this study are available within the article or can be made available upon reasonable request.
